# Supplementary figures and images for: Using the First-Eye Back-Calculated Effective Lens Position to Improve Refractive Outcome of the Second Eye
Source: J Clin Med. 2022 Dec 26;12(1):184. doi: 10.3390/jcm12010184 (PMC9820906; doi:10.3390/jcm12010184)

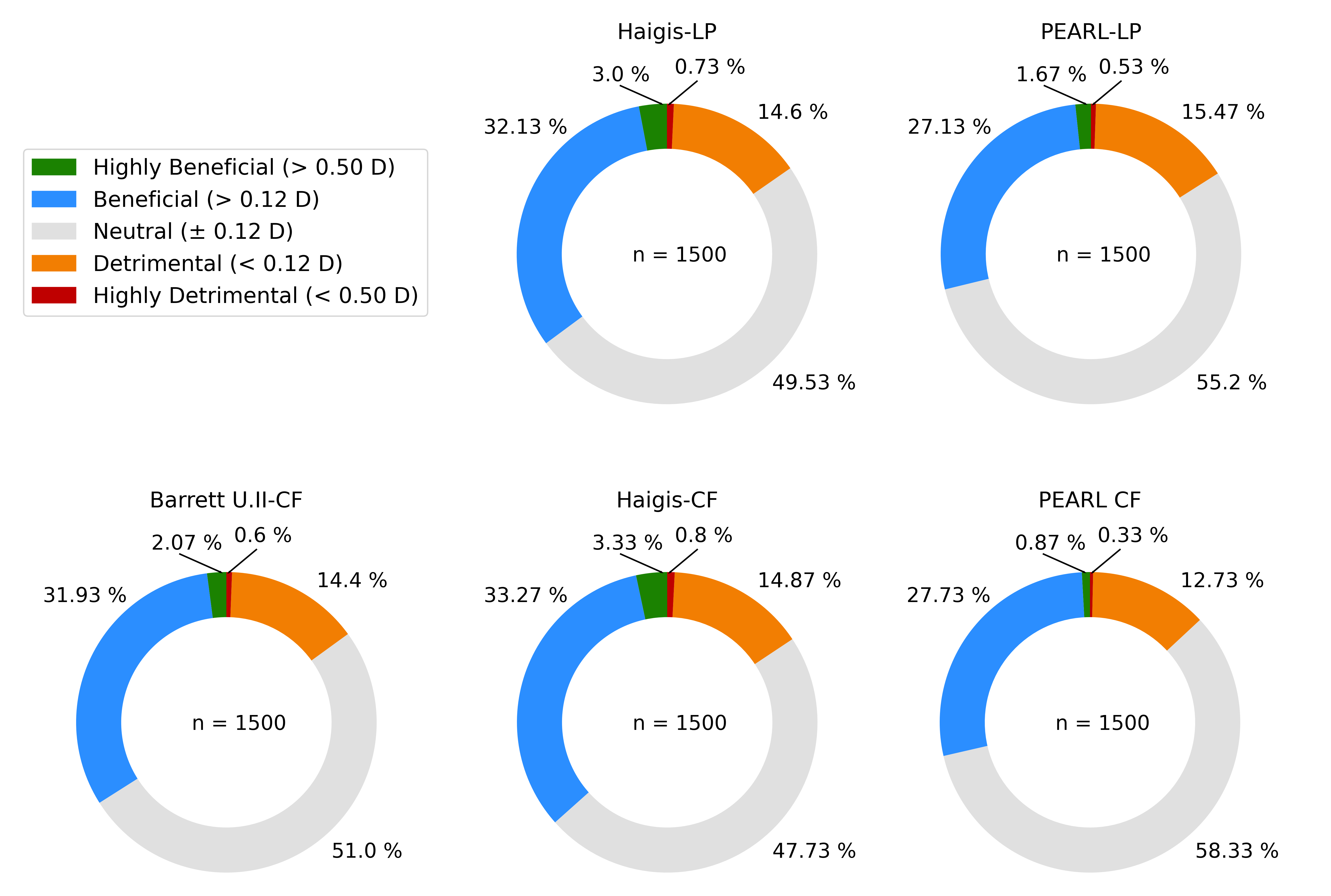

Supplement: Supplementary file 1 [file jcm-12-00184-s001.zip › jcm-2085180-supplementary.png]
